# Comparative Evaluation of Phenolic Composition and Antioxidant Activities of *Gastrodia elata* Rhizome Cultivated in the Indoor Facility and Outdoor Field: Effects of Cultivation Type, Growth Stage and Harvest Season

**DOI:** 10.3390/foods15172985

**Published:** 2026-08-25

**Authors:** Hyun Jin Choi, Ye Seul Kwon, Yoseph Asmelash Gebru, Young-Hoi Kim, Eun-Suk Lee, Chang-Su Kim, Han-Seok Choi

**Affiliations:** 1 Department of Food Science and Technology, Jeonbuk National University, Jeonju 54896, Republic of Korea; chj00614@sookmyung.ac.kr (H.J.C.); yeseul961116@gmail.com (Y.S.K.); 2 Department of Animal Science and Technology, Chung-Ang University, Anseong 17546, Republic of Korea; yagebru@gmail.com; 3 Department of Agriculture & Fisheries FoodTech, Korea National University of Agriculture and Fisheries, Jeonju 54874, Republic of Korea; sangde1307@naver.com; 4 Jeonbuk State Agricultural Research & Extension Services, Iksan 54591, Republic of Korea; es10014@korea.kr (E.-S.L.); florigen5329@korea.kr (C.-S.K.)

**Keywords:** *Gastrodia elata*, indoor facility, outdoor field, cultivation type, growth stage, harvest season, phenolic composition, antioxidant activities

## Abstract

*Gastrodia elata* rhizome (GER), long used medicinally and as food in East Asia, is now produced via outdoor field (OFC) or indoor facility (IFC) cultivation. This study evaluated phenolic composition and antioxidant properties of GER by cultivation method, growth stage, and harvest season. Seven compounds were quantified by HPLC; parishin A, parishin B, parishin E, and *p*-hydroxybenzyl methyl ether (*p*-HBME) were the major constituents. Among mature samples, their total content ranged from 13.21 to 18.33 mg/g DW in OFC-grown GER and was 12.15 mg/g DW in IFC-grown GER, while immature GER contained up to 26.16 mg/g DW. However, IFC and OFC samples originated from different regions, so differences cannot be attributed solely to cultivation method. Spring-harvested GER had a higher total content of the seven compounds than autumn-harvested GER (20.67 ± 0.17 and 12.01 ± 0.19 mg/g DW, respectively) and stronger DPPH, FRAP, and ABTS activities, whereas total phenolic content (TPC) was higher in autumn. Seasonal comparisons reflect extraction replicates of composite samples, not independent biological replicates. ABTS activity was positively correlated with gastrodin, 4-hydroxybenzyl alcohol (4-HBA), *p*-HBME, parishin A, and total flavonoid content (TFC). PCA explained 71.5% of total variance (PC1–PC2), supporting discrimination among GER samples by phenolic and antioxidant profiles.

## 1. Introduction

*Gastrodia elata* Blume (GER) is a shade perennial plant that belongs to the Orchidaceae family. Due to its lack of chlorophylls, it cannot perform photosynthesis on its own and therefore grows by obtaining nutrients through a symbiotic relationship with the fungus *Armillaria* sp. [1,2,3]. The GER, called Chunma in Korea and Tianma in China, has traditionally been used as both a herbal medicine and food material [4,5,6]. Pharmacologically, it has been utilized as a crude drug for the treatment and improvement of neurological or cerebrovascular disorders such as hypertension, headache, dizziness, convulsions, pain, sedation, and epilepsy [7,8,9,10]. Moreover, recent in vitro and in vivo experimental models and clinical studies have demonstrated that GER possesses neuroprotective and cognitive-enhancing effects on conditions such as Alzheimer’s disease, epilepsy, convulsions, cerebral infarction, hypertension, and dementia [11,12,13,14]. Moreover, it has been reported to exhibit memory-improving and anti-dementia effects [15,16,17], anticonvulsant effects [18,19], anti-inflammatory effects [20,21], and antioxidant activities [22,23].

To date, over 630 chemical compounds have been identified in GER, including phenolic compounds, polysaccharides, glycosides, organic acids, and sterols [12,24]. GER’s distinctive bioactivities are closely associated with its unique constituents, mainly the structurally diverse and quantitatively abundant phenolic compounds in the species [5]. Among these, phenolic glycosides, i.e., parishin A (tris[4-(β-D-glucopyranosyloxy)benzyl]citrate), parishin B, parishin C, parishin E, gastrodin and their metabolite 4-hydroxybenzyl alcohol (4-HBA) are abundant in GER and have been extensively studied for pharmacological activities [25,26,27].

GER was traditionally collected from the wild. However, its natural population has recently been depleted due to environmental changes including overharvesting and overdevelopment of forests. This led to the development of artificial cultivation methods, such as OFC, which were then distributed to farmers; the majority of GER currently available in the market is produced through the OFC method. However, recent issues such as climate fluctuations, degeneration of fungal inoculum caused by vegetative propagation, and continuous cropping disorders have led to yield reduction and quality deterioration, emphasizing the need for more advanced cultivation systems. In response, an IFC method has been developed to maintain optimal environmental conditions for stable production and improved quality. However, research on the phenolic composition and antioxidant activity of GERs cultivated via IFC and OFC methods remains limited. The yield per unit area and the composition of major chemical constituents of produced GER are greatly influenced by various factors, including geographical climate, soil characteristics, cultivation conditions, planting techniques, and interactions with symbiotic fungi.

Therefore, this study aimed to compare the composition of phenolic compounds and antioxidant activities of GER produced under the two different cultivation methods and to investigate the effects of growth stage and harvest season on the phenolic compounds profile and antioxidant properties of GER. The results of this study are expected to contribute to improving cultivation conditions and determining the optimal harvest time, thereby improving the potential of GER as a medicinal herb and functional food material.

## 2. Materials and Methods

### 2.1. Materials

**Figure 1:**
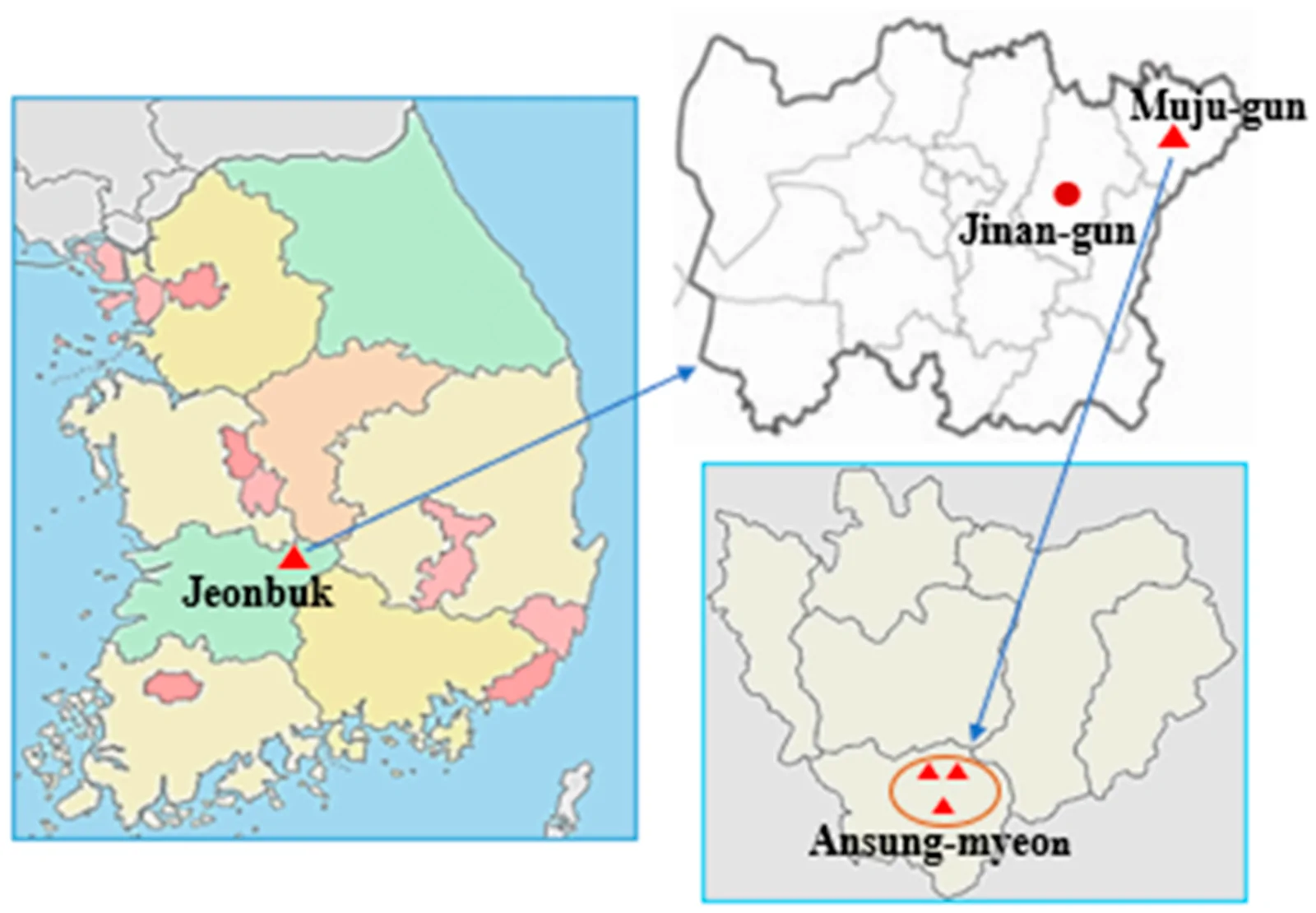
Geographical location of cultivation sites of GERs by the OFC (▲) and IFC (●) methods.

Cultivation of GER by IFC was conducted at Jinan Medicinal Resources Research Institute (Jinan-eup, Jinan-gun, Jeonbuk, Republic of Korea) between 2022 and 2025, while OFC trials were carried out at three different farms in Ansung-myeon (Muju-gun, Jeonbuk, Republic of Korea), which is the main cultivation region of GE in Korea. The cultivation sites are provided in Figure 1. These locations were mountainous regions with annual average temperature, precipitation and altitude of 20 °C, 1300 mm and 500 m, respectively. IFC seedlings planted in April 2022 were harvested in April (spring) and October (autumn) of 2024, and plants set in April 2023 were harvested after two years (April 2025). OFC seedlings planted in April 2023 at three different farms were similarly harvested after two years (April 2025).

**Figure 2:**
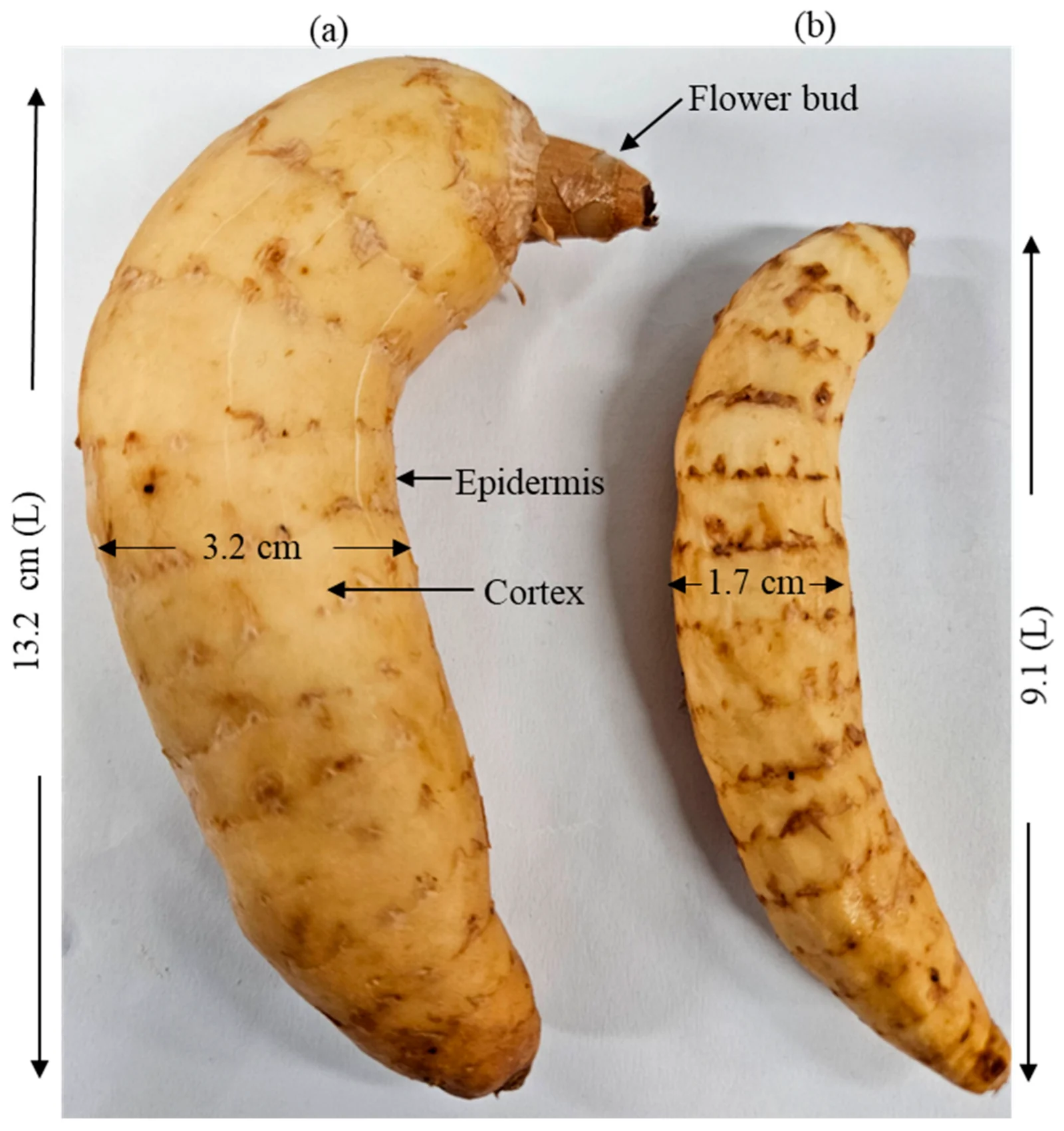
Morphology of mature (**a**) and immature GERs (**b**) produced by IFC. Average weight per piece: (**a**), 60.5 ± 8.3 g (*n* = 10); (**b**), 16.2 ± 5.2 g (*n* = 10).

For the IFC experiment, a rain-shielded facility (4.8 m wide × 7.5 m long × 5.5 m high) was installed and covered with a 0.1 mm-thick polyethylene (PE) film. A shade cloth (95% shading) capable of blocking sunlight was installed from late June to early September. The GER planting boxes were standardized to a specific size (width 32 cm × length 44 cm × height 18 cm) with five drainage holes (30 cm diameter per hole). For cultivation logs, two pieces of dry oak wood (diameter 10–12 cm × length 20 cm) were placed per box. *Armillaria gallica* spawn, cut into 8 pieces per bottle (1 L), was inoculated at 4 pieces per box. Cultivation substrate was then applied over the logs to a depth of 10 cm. The potting soil was a mixture of peat moss and perlite in a ratio of 7:3 (*v*/*v*). Irrigation was performed after covering the soil. Moisture management of the culture medium was maintained at –30 kPa during the tuber formation stage and −50 kPa during the tuber enlargement stage. The temperature within the indoor facility was controlled according to distinct phases: mycelium establishment (20 ± 1 °C), tuber formation (25 ± 1 °C), tuber enlargement (15 ± 1 °C), and dormancy (5 ± 1 °C). The OFC was conducted from the first half of 2022 to the first half of 2025 at three randomly selected farms in Ansung-myeon (Muju-gun, Jeonbuk, Republic of Korea), as shown in Figure 1. The cultivation site had ridges 100 cm wide, ridge heights of 50 cm, and furrow depths of 90 cm. Seed rhizomes for planting (10–20 g each) were selected from rhizomes cultivated in Ansung-myeon (Muju-gun), and planted at a rate of 120–150 kg per unit (10 a). Dried oak logs (diameter 12–15 cm × length 30–35 cm) were cut and used at 5 logs per ridge, totaling 4500–4800 logs per unit (10 a). The *A. gallica* spawn used was identical to the one employed in the IFC method. The spawn of *A. gallica* was obtained from the Jungbu Microbiology Research Institute (Iwon-myeon, Ockcheon-gun, Chungbuk, Republic of Korea). The soil type at all three selected OFC sites was sandy loam, with organic matter content of around 0.5%. Other cultivation site management practices followed previously reported methods [28,29]. GERs harvested in April 2025 after cultivation by the two methods were categorized as mature or immature based on whether flower buds were formed and the rhizome morphology (weights and dimensions), as depicted in Figure 2. The detailed specifications of the samples used in this study are listed in Table 1.

### 2.2. Sample Preparation

Each harvested sample was selected only if it showed no external wounds or signs of decay, as illustrated in Figure 2. For each condition, three or four individual rhizomes with similar weights and sizes (approximately 3 kg per sample in total) were washed with tap water, cut into 1 cm pieces, and pooled into a single composite sample. The composite sample was freeze-dried (FD5508, IlshinBioBase Co., Dongducheon-si, Gyeonggi-do, Republic of Korea), finely ground using a mill (SFM-555SP, Shinil Industrial Co., Ltd., Eumseong-gun, Chungnam, Republic of Korea), and stored at –20 °C for later use.

### 2.3. Reagents

Gallic acid, rutin, 4-HBA, 2,2-diphenyl-1-picrylhydrazyl (DPPH), 6-hydroxy-2,5,7,8-tetramethylchromane-2-carboxylic acid (Trolox), 2,2′-azino-*bis*(3-ethylbenzothiazoline-6-sulfonic acid) diammonium salt (ABTS), 2,4,6-tri(2-pyridyl)-*s*-triazine (TPTZ) were purchased from Sigma-Aldrich (St. Louis, MO, USA). Gastrodin and parishins (A, B, C, and E), with purities of ≥98%, were purchased from Wuhan ChemFaces Biochemical Co., Ltd. (Wuhan, Hubei Province, China). The thin layer chromatography (TLC) plates (0.25 mm), silica gel (70–230 mesh) for column chromatography, deuterated methanol (CD_3_OD), and tetramethylsilane (TMS) were purchased from Merck KGaA (Darmstadt, Germany). Toyopearl HW-40S polymer was purchased from Tosoh Corp. (Tokyo, Japan). Methanol (MeOH) of High-performance liquid chromatography (HPLC) grade was purchased from Advantor Performance Materials Korea (Suwon-si, Gyunggi-do, Republic of Korea). Deionized water was prepared using a water purification system (Model New Human Power I, Human Corp., Songpa-gu, Seoul, Republic of Korea). Other general reagents were purchased from Daehan Science Co., Ltd. (Wonju-si, Gangwon-do, Republic of Korea) as analytical-grade reagents.

### 2.4. Isolation and Identification of Major Phenolic Compounds from GERs Produced by IFC

#### 2.4.1. Semi-Preparative Extraction

First, 1.5 L of 70% methanol aqueous solution was added to 500 g of powdered GER sample (mature IFC harvested in October 2024). The mixture was then sonicated at room temperature for 30 min followed by centrifugation at 5000× *g* for 20 min. The residue was extracted twice more using the same method. The three resulting supernatants were concentrated under reduced pressure at 50 °C to yield 87 g (yield 10.2%) of the concentrate. Then, 40 g of the concentrate was suspended in 300 mL of distilled water, and sequentially extracted with *n*-hexane, ethyl acetate, and water-saturated *n*-butanol (300 mL × 3) according to Otsuka’s method [30]. Each fraction was concentrated under reduced pressure, yielding an *n*-hexane fraction (0.66 g), an ethyl acetate fraction (5.42 g), and a butanol fraction (0.73 g). Furthermore, the water layer remaining after the water-saturated *n*-butanol extraction was concentrated under reduced pressure, dissolved in a small amount of distilled water, and adsorbed onto a pre-activated Amberlite XAD-2 column (4.5 × 40 cm). Water-soluble compounds were removed by washing with distilled water (approximately 1 L). Subsequently, the compounds adsorbed onto the resin were eluted and recovered with methanol (1 L), then concentrated under reduced pressure to yield 1.06 g of concentrate.

#### 2.4.2. Isolation of Compounds

The *n*-hexane and ethyl acetate fractions obtained by solvent partitioning were combined, and a portion (5.0 g) was dissolved in MeOH and separated by silica gel column chromatography (35 × 4.5 cm) using a stepwise *n*-hexane–ethyl acetate gradient (95:5 → 90:10 → 80:20 → 60:40 → 50:50 → 40:60 → 0:100, *v*/*v*; 500 mL each), collecting 30 mL fractions that were concentrated under reduced pressure. Each concentrate was monitored by TLC (CHCl_3_-MeOH-H_2_O, 65:35:10, *v*/*v*/*v*, lower phase), with spots visualized under UV (254 nm), and by spraying with 10% sulfuric acid followed by heating at 110 °C for 10 min. Compound 1 (1.9 g, white powder) was obtained from the fraction eluted with 60:40 (tubes 38–51), and compound 2 (0.3 g) from the fraction eluted with 40:60 (tubes 70–84).

The aqueous layer was applied to an Amberlite XAD-2 column and eluted with MeOH, and the resulting concentrate (4.1 g) was dissolved in MeOH and further separated by silica gel column chromatography (30 × 4.5 cm) using a stepwise CHCl_3_-MeOH-H_2_O gradient (90:10:1 → 80:20:2 → 70:30:3 → 65:35:10 [lower phase] → 60:40:10 → 0:50:50, *v*/*v*; 500 mL each), collecting 30 mL fractions. Fractions eluted with 65:35:10 (lower phase) were combined and concentrated under reduced pressure, and fractions eluted with 60:40:10 (tubes 67–80), containing compounds 3 and 4, were further purified on a Toyopearl HW-40 column (35 × 2.5 cm) with 70% MeOH to yield compounds 3 (43 mg) and 4 (21 mg), respectively.

The chemical structures of the isolated compounds were elucidated based on UV–Vis spectroscopy, GC-MS, ESI-UPLC-QTOF-MS (positive and negative ion modes), and ^1^H and ^13^C NMR spectral data.

### 2.5. Analysis of Phenolic Compounds by HPLC

#### 2.5.1. Extraction

After 5 mL of 70% MeOH was added to 0.2 g of freeze-dried powdered composite sample, the mixture was sonicated twice for 30 min each at room temperature and was then centrifuged (6000× *g*, 20 min; Himac centrifuge, type CR22N, Koki Holdings Co., Ltd., Ibaraki, Japan). Subsequently, a portion of the supernatant was filtered through a 0.45 µm microsyringe filter (Sartorius AG, Göttingen, Germany) and analyzed by high-performance liquid chromatography (HPLC).

#### 2.5.2. HPLC Analysis

HPLC analysis was performed using an HPLC system (Waters, Milford, MA, USA) consisting of a Waters 2690 separation module and a Waters 996 photodiode array detector (PDA). The column used was a Zorbax Eclipse XDB-C18 column (4.6 mm × 250 mm, 5 µm; Agilent Technologies, Inc., Santa Clara, CA, USA). The mobile phase consisted of deionized water containing 0.1% formic acid (A) and methanol containing 0.1% formic acid (B), and analysis was performed in gradient mode. The gradient conditions were A:B = 97:3 (0–5 min), 90:10 (5–10 min), 80:20 (10–15 min), 60:40 (15–30 min), 30:70 (30–35 min), 0:100 (35–40 min) and 97:3 (40–45 min). The flow rate was set at 1.0 mL/min. Detection was performed using a photodiode array (PDA) detector at 220 nm. Each component was quantified using external calibration with serially diluted standards. All experiments were performed in triplicate, and the data were expressed as the mean ± standard deviation (mg/g DW). The maximum absorption wavelengths (UV maxima), calibration curves, linear ranges, and coefficients of determination (R^2^) for the seven compounds are presented in Table 2.

### 2.6. Determination of Total Phenol Content (TPC) and Total Flavonoid Content (TFC)

Total phenolic content (TPC) was measured using the Folin–Denis method [31]. Briefly, 2150 μL of 2% sodium carbonate solution was added to 100 μL of the sample extract. After reacting for 2 min, 250 μL of 50% Folin–Ciocalteu reagent was added, mixed, and allowed to react for 30 min. The absorbance was measured at 725 nm using a UV-Vis spectrophotometer. The content of total phenolic compounds was determined from a calibration curve prepared separately using gallic acid and expressed as mg/g DW. Total flavonoid content (TFC) was measured according to the method of Zhishen et al. [32]. Briefly, 250 μL of the sample extract was mixed with 1.6 mL of distilled water and 75 μL of 5% NaNO_2_ solution, then allowed to stand for 5 min. Next, 75 μL of a 10% AlCl_3_·6H_2_O solution was added and maintained for 1 min. Then, 500 μL of a 1 M NaOH solution was added, and the absorbance was measured at 510 nm using a UV-Vis spectrophotometer. The total flavonoid content was determined from a calibration curve prepared separately using rutin and expressed as mg/g DW.

### 2.7. Antioxidant Activities

DPPH radical scavenging activity was measured with a slight modification of the method of Blois [33]. Briefly, 100 μL of sample extract was mixed with 500 μL of 0.1 M Tris-HCl buffer and 80 μL of 500 μM DPPH in ethanol, and the mixture was allowed to stand at room temperature for 20 min. The absorbance was then measured at 517 nm using a UV-Vis spectrophotometer. DPPH radical scavenging capacity was calculated from a calibration curve prepared with 1.5 mM Trolox and expressed as mM Trolox equivalent antioxidant capacity (TEAC)/g DW, and TEAC by the DPPH assay exhibited linearity in the range of 0–105 μM (y = 0.3564x + 2.9646, R^2^ = 0.9963).

ABTS radical scavenging activity was determined according to the method of Thaipong et al. [34] with slight modification. A 7.4 mM ABTS solution and a 2.6 mM potassium persulfate solution were mixed in equal volumes (1:1) and incubated for 12 h at room temperature in the dark. The resulting solution was diluted with ethanol to an absorbance of 1.1 (±0.02) at 734 nm. Then, 200 μL of sample extract was added to 2800 μL of the diluted ABTS solution, incubated at 30 °C for 2 h, and the absorbance was measured at 734 nm. Radical scavenging capacity was calculated from a calibration curve prepared with 1.5 mM Trolox and expressed as mM TEAC/g DW, and TEAC by the ABTS assay exhibited linearity in the range of 0–105 μM (y = 0.1416x + 1.1526, R^2^ = 0.9963).

FRAP reducing activity was measured using a modified method of Benzie and Strain [35]. A 300 mM acetate buffer (pH 3.6), 10 mM TPTZ in 40 mM HCl, and 20 mM FeCl_3_·6H_2_O were mixed in a 10:1:1 (*v*/*v*/*v*) ratio and pre-incubated at 37 °C for 15 min. Sample extract (150 μL) was mixed with 2850 μL of the pre-reacted FRAP reagent, incubated for 15 min, and the absorbance was measured at 593 nm. Calibration curves were constructed using Trolox (15–1500 μM), FRAP values were expressed as mM TEAC/g DW, and TEAC by the FRAP assay exhibited linearity in the range of 0–225 μM (y = 0.013x + 0.081, R^2^ = 0.9984).

### 2.8. General Experimental Conditions

Gas chromatography–mass spectrometry (GC-MS) analysis was performed using a Trace 1310 gas chromatograph connected to an ISQ single quadrupole mass spectrometer (Thermo Fisher Scientific Inc., Waltham, MA, USA) operating in the EI mode at 70 eV, fitted with a DB-5MS fused silica capillary column (30 m × 0.25 mm, 0.25 µm film thickness). The column temperature was programmed from 50 °C to 230 °C at 2 °C per minute and then kept constant at 230 °C for 5 min. The injector and ion source temperatures were 250 °C. Other conditions of GC-MS were carrier gas (helium) flow, 1.0 mL/min; split ratio, 1:10; ionization voltage, 70 eV; and mass scan range, 25–500 *m*/*z*. NMR spectra were recorded on a JEOL model JNM−ECA 600 FT−NMR spectrometer (Akishima, Tokyo, Japan) at ^1^H-NMR (600 MHz), and ^13^C-NMR (150 MHz) with CD_3_OD and TMS as a solvent and internal standard, respectively.

### 2.9. Statistical Analysis

As described in Section 2.2, each condition consisted of a composite sample pooled from three or four individual rhizomes, which was then extracted three or four times (extraction replicates); unless otherwise indicated, results are expressed as the mean ± SD of these extraction replicates rather than independent biological replicates. Statistical analysis was performed using SPSS software (ver. 12.0, SPSS Inc., Chicago, IL, USA). Differences between two groups were analyzed using Student’s *t*-test, whereas comparisons among three or more groups were performed using one-way analysis of variance (ANOVA) followed by Duncan’s multiple range test. Values with *p* < 0.05 were considered statistically significant. Pearson correlation coefficients were calculated using Python (version 3.13; Python Software Foundation, Wilmington, DE, USA) to evaluate the relationships between bioactive compounds and antioxidant activities. Principal component analysis (PCA) was also conducted using Python (version 3.13), and the results were visualized as a PCA biplot. The first two principal components (PC1 and PC2) were used to explain the major variation within the dataset.

## 3. Results

### 3.1. Comparison of Phenolic Profiles in GER by HPLC

**Figure 3:**
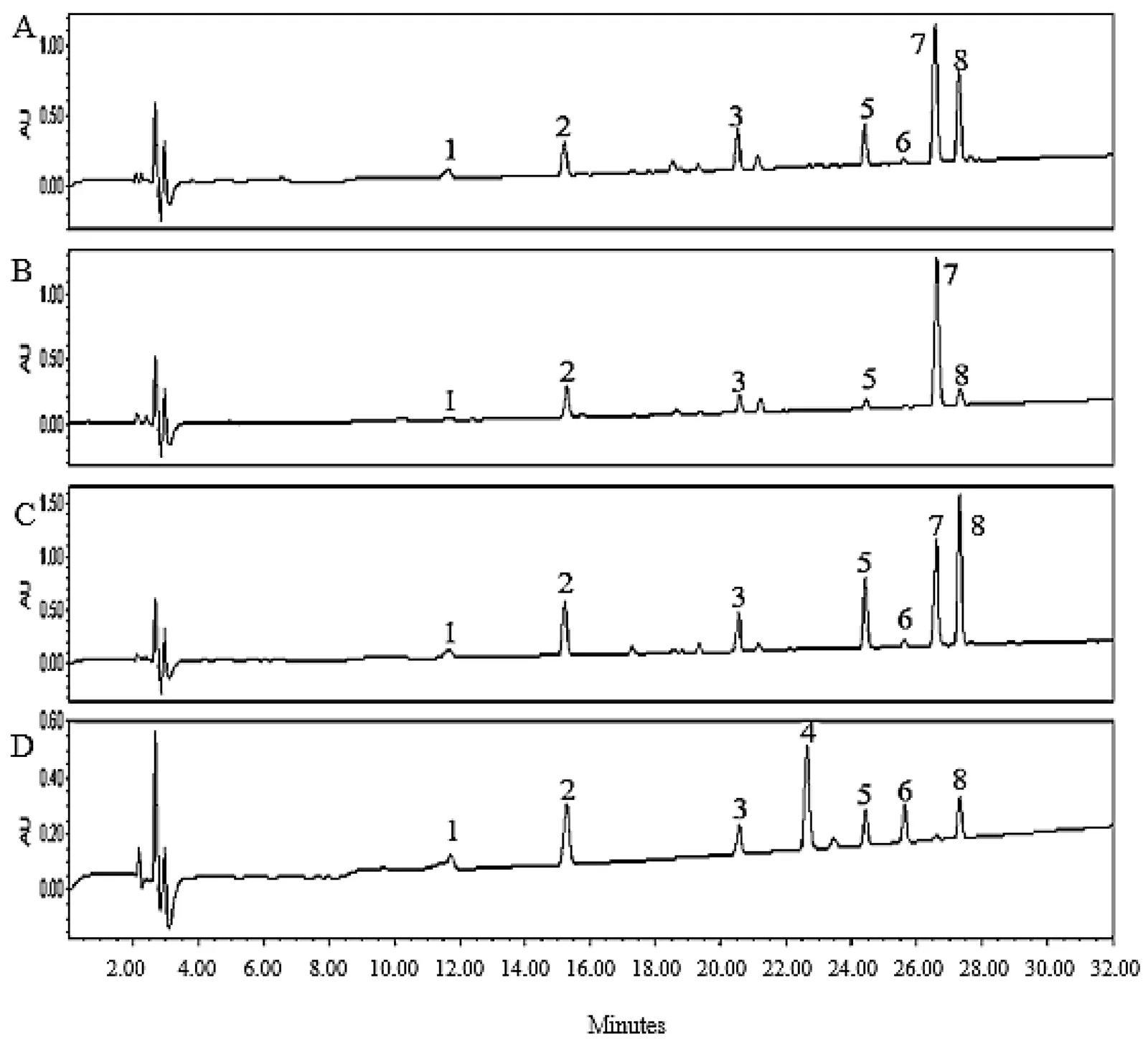
Comparison of HPLC profiles of phenolic compounds in mature GER cultivated by two different cultivation methods. Two years of cultivation in indoor facility and harvested in spring (**A**) and autumn (**B**). Two years of cultivation in outdoor facility and harvested in spring (**C**). Standard mixture of parishin derivatives (**D**). 1, gastrodin; 2, 4-hydroxybenzyl alcohol (gastrodigenin); 3, parishin E; 4, *p*-hydroxybenzaldehyde; 5, parishin B; 6, parishin C; 7, unknown; 8, parishin A.

Among the bioactive constituents in GER, phenolic compounds including 4-HBA, gastrodin, and parishin derivatives have received particular attention. GER samples produced by the IFC method and harvested in spring (Figure 3A) and autumn (Figure 3B), as well as GER produced by the conventional OFC method (Figure 3C), were extracted using 70% methanol and analyzed by HPLC. When compared with authentic standards, the IFC-grown GER showed clear peaks corresponding to parishin A, B, C and E, which are major phenolic compounds previously reported in GER, as well as their hydrolysis product, 4-HBA, which was detected as one of the predominant constituents. In contrast, gastrodin was detected only in trace amounts. Furthermore, in GER produced by the OFC method, relatively large peaks were observed, which might have been attributed to the presence of PA (peak 8), PB (peak 5), PE (peak 3), and 4-HBA (peak 2). The consistently small peak of gastrodin in both of the cultivation methods is suggested to indicate its low content in GER. Notably, a prominent peak corresponding to an unknown compound (peak 7) was detected, and this tendency was especially pronounced in IFC-grown samples harvested in autumn. Based on this, we attempted to elucidate the structures of the major components detected, including the unknown compound (peak 7), by conducting chromatographic separations.

### 3.2. Isolation and Identification of Major Compounds from GER Produced by IFC

GER cultivated by the IFC method and harvested in autumn was extracted with 70% MeOH. The resulting extract was subjected to column chromatography using silica gel and Toyopearl HW-40S, yielding four isolated compounds (peak no. 2, 5, 7, and 8). The compound of peak no. 2 in Figure 3 was isolated as a pale amorphous yellow powder with UV (λ_max_ 223.0, 273.6 nm). Analysis by GC-MS revealed a molecular ion peak (M^+^) at m/z 124 (C_7_H_8_O_2_). This peak matched the mass spectrum of 4-hydroxybenzyl alcohol (4-HBA) in NIST Chemistry WebBook (SRD 69, National Institute of Standards and Technology, USA). Furthermore, ^1^H-NMR spectrum (600 MHz, CD_3_OD) exhibited signals at δ 7.17 (2H, d, *J* = 8.4 Hz, H-2, 6), 6.75 (2H, d, *J* = 8.4 Hz, H-3, 5), and 4.48 (2H, s, H-7). The ^13^C-NMR spectrum (150 MHz, CD_3_OD) showed resonance at δ 158.0 (C, C-4), 133.4 (C, C-1), 129.9 (CH, C-2, 6), 116.1 (CH, C-3, 5), and 65.1 (CH_2_, C-7). The detailed assignments are listed in Table 3. The UV profile, GC-MS, and NMR data confirmed this compound was 4-HBA, with all spectral characteristics consistent with previously reported values in the literature [36]. Although 4-HBA occurs in various plant species, it is particularly abundant in GER. This compound is a hydrolysis product and metabolic intermediate of parishins and gastrodin and is recognized as one of the major bioactive constituents of GER [37,38].

**Figure 4:**
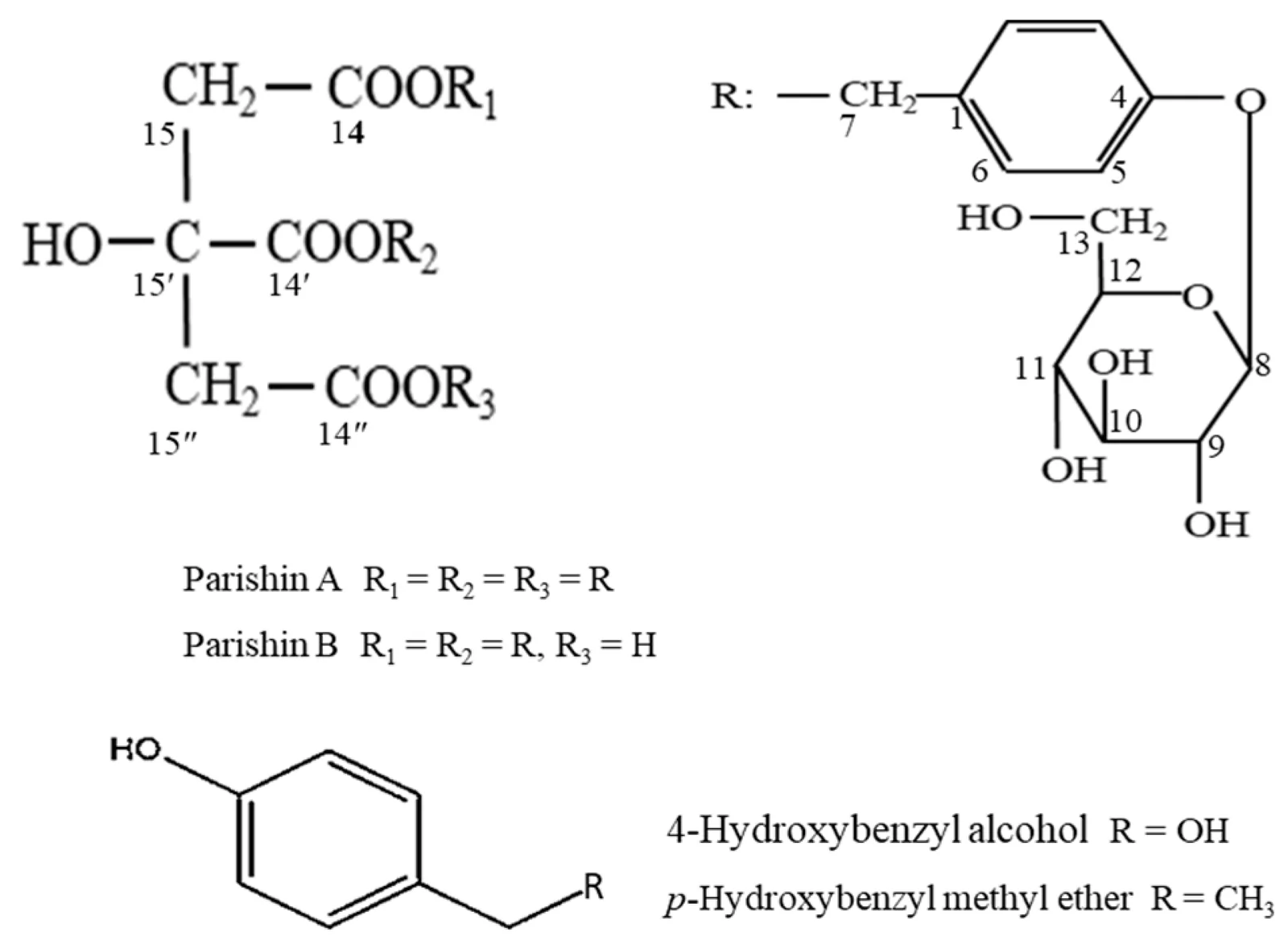
Proposed chemical structures of major phenolic compounds identified in GER produced using the IFC method.

The compound corresponding to peak 5 was isolated as a white amorphous powder. Its UV spectrum exhibited absorption maxima at 221.7 and 269.2 nm. The negative-QTOF-MS spectrum showed a deprotonated molecular ion at m/z 727.2033 [M−H]^−^, corresponding to the molecular formula of C_32_H_40_O_19_ (molecular mass 728.65). The ^1^H-NMR spectrum (600 MHz, CD_3_OD) of compound 5 revealed two *p*-disubstituted benzene rings with signals at δ 7.26 (2H, d, *J* = 8.1 Hz, H-2, 6), 7.21 (2H, d, *J* = 8.1 Hz, H-2′, 6′), 7.08 (2H, d, *J* = 8.3 Hz, H-3, 5) and 7.02 (2H, d, *J* = 8.3 Hz, H-3′, 5′). Two benzylic methylene groups were observed at δ 4.90 (2H, br. s, H-7a, 7b) and 4.87 (2H, br. s, H-7′a, 7′b), along with two anomeric proton signals at δ 4.89 (2H, d, J = 7.5 Hz, H-8 and H-8′), indicating the presence of two gastrodin moieties in the molecule. The ^13C-NMR spectrum (150 MHz, CD_3_OD) of peak no. 5 showed three carbonyl carbon signals at δ 175.1 (C-14′) and 171.2 (C-14 and C-14″); aromatic carbon signals at δ 159.2 (×2, C-4 and C-4′), 131.0 (×6, C-1, C-1′, C-2, C-2′, C-6, and C-6′), and 117.8 (×4, C-3, C-3′, C-5, and C-5′); signals attributable to two glucose units at δ 102.3 (×2, C-8 and C-8′), 78.1 (×2, C-10 and C-10′), 78.0 (×2, C-12 and C-12′), 74.9 (×2, C-9 and C-9′), 71.4 (×2, C-11 and C-11′), and 62.5 (×2, C-13 and C-13′); benzylic methylene carbon signals at δ 68.2 (C-7′) and 67.3 (C-7); and citrate-derived carbon signals at δ 74.8 (C-15′) and 44.9 (×2, C-15 and C-15″). Based on the above results, compound 4 was identified as 1,2-bis [4-(β-D-glucopyranosyloxy)benzyl] citrate, namely parishin B (Figure 4). Parishin B was previously identified in GER [39,40,41] and more recently in *M. tricuspidata* twig [42]. The parishin compounds and their metabolites gastrodin and 4-HBA have been recognized as important bioactive constituents in GER and exhibit anticonvulsant, analgesic, sedative, hypnotic, nootropic, and anti-brain aging activities in traditional Chinese medicine [13,27].

Meanwhile, the UV profile (λmax 223.0, 272.5 nm) of the unknown compound (peak no. 7) was highly similar to that of 4-HBA (λ_max_ 223.0, 273.6 nm). Analysis by GC-MS revealed a molecular ion peak at m/z 138 (C_8_H_10_O_2_). Furthermore, ^1^H-NMR data (600 MHz, CD_3_OD) showed proton signals due to the presence of an aromatic ring at δ 7.12 (d, 2H, *J* = 9.0 Hz, H-2, 6) and 6.73 (d, 2H, *J* = 8.4 Hz, H-3, 5), along with additional signals at δ 4.30 (s, 2H) and 3.29 (s, 3H) as shown in Table 3.

These results suggest that peak no. 7 corresponds to a compound bearing one methylene and a methoxy substituent attached to the aromatic ring. Additionally, the ^13^C-NMR (150 MHz, CD_3_OD) measurement results showed signals at δ 130.79 (×2, C-2, 6), 116.24 (×2, C-3, 5), 129.08 (C-1), 158.33 (C-4), 75.53 (C-7), and 57.79 (C-8). The DEPT spectrum indicated four methine carbons, one methylene carbon, and one methyl carbon. Based on these results, the compound of peak 7 was identified as *p*-hydroxybenzyl methyl ether (4-(methoxymethyl)phenol). Although this compound has been previously described as a minor constituent in GER, another report indicated that it might be present in significant quantities [41]. It has been reported to exhibit learning and memory-enhancing effects in vivo [43].

The compound corresponding to peak no. 8 was obtained as a white amorphous powder. The UV spectrum showed absorption maxima at 221.7 and 269.2 nm. The negative and positive ESI-MS spectra showed ion peaks at *m*/*z* 995.3 [M−H]^−^ and 1019.3 [M + Na]^+^, respectively, corresponding to the molecular formula C_45_H_56_O_25_ (molecular mass 996.92). The ^1^H-NMR spectrum (600 MHz, CD_3_OD) of compound 3 indicated the presence of three *p*-disubstituted benzene rings at δ 7.26 (4H, d, *J* = 7.8 Hz, H-2, 2″, 6, 6″), 7.16 (2H, d, *J* = 8.4 Hz, H-2′, 6′), 7.07 (4H, d, *J* = 8.7 Hz, H-3, 3″, 5, 5″), and 7.04 (2H, d, *J* = 7.8 Hz, H-3′, 5′). Three benzylic methylene groups gave signals at δ 4.98 (2H, s, H-7′) and 4.88 (4H, s, H-7, 7″). Three anomeric proton signals were observed at δ 4.91 (3H, d, J = 7.2 Hz, H-8, H-8′, and H-8″), indicating the presence of three β-D-glucopyranosyl moieties. Additionally, two benzylic methylene proton signals were detected at δ 2.94 (2H, d, *J* = 15.6 Hz, H-15a, 15″a) and 2.77 (2H, d, *J* = 15.0 Hz, H-15b, 15″b). The ^13C-NMR spectrum (150 MHz, CD_3_OD) of the compound corresponding to peak no. 8 exhibited three carbonyl carbon signals at δ 174.5 (C-14′) and 171.1 (×2, C-14 and C-14″); signals attributable to three glucose units at δ 102.3 (×3, C-8, C-8′, and C-8″), 78.2 (×3, C-10, C-10′, and C-10″), 77.9 (×3, C-12, C-12′, and C-12″), 75.0 (×3, C-9, C-9′, and C-9″), 71.4 (×3, C-11, C-11′, and C-11″), and 62.5 (×3, C-13, C-13′, and C-13″); aromatic carbon signals at δ 159.2 (×3, C-4, C-4′, and C-4″), 131.2 (×6, C-2, C-2′, C-2″, C-6, C-6′, and C-6″), 131.2 (×2, C-1 and C-1″), 130.8 (C-1′), and 117.9 (×6, C-3, C-3′, C-3″, C-5, C-5′, and C-5″); benzylic methylene carbon signals at δ 68.4 (C-7′) and 67.5 (×2, C-7 and C-7″); and methylene carbon signals of the citrate moiety at δ 44.9 (×2, C-15 and C-15″). Based on comparison with previously reported spectroscopic data [36], the compound corresponding to peak no. 8 was identified as tris[4-(β-D-glucopyranosyloxy)benzyl]citrate, namely parishin A (Figure 4). This compound has previously been reported from GER [37,38], *Vanda parishii* [44] and *Maclura tricuspidata* twig [42].

### 3.3. Composition of Phenolic Compounds

#### 3.3.1. Effect of Cultivation Method and Growth Stage

GER has traditionally been harvested from natural forest habitats for medicinal or food use. However, declines in wild populations caused by various environmental and anthropogenic factors have led to a shift towards artificial cultivation methods, such as forest-edge cultivation and OFC. More recently, the IFC method, i.e., an intensive and rapid cultivation system that enables high-yield in short-term production time by artificially controlled environmental conditions such as temperature and humidity, has been developed and is becoming increasingly widespread. The IFC method involves cultivating GER inside facilities constructed by covering a plastic greenhouse with a shade cloth. This system enables precise water management through irrigation control and provides a stable cultivation environment with improved temperature regulation. As with many plant species, the composition and content of phytochemicals in GER vary depending on genetic variation, cultivation method, geographical origin, meteorological conditions, harvest time, and plant part. In this study, the contents of seven components were quantified by HPLC in mature and immature GER cultivated using the IFC and OFC methods (Table 4). In mature GER produced by the IFC method, *p*-HBME was found to be present at the highest level (4.95 ± 0.07 mg/g DW), followed by parishin E (3.44 ± 0.05 mg/g DW) and parishin A (1.25 ± 0.01 mg/g DW). Similarly, in mature GER produced by the OFC method across three farm locations, *p*-HBME was the predominant component (4.66 ± 0.04–6.65 ± 0.09 mg/g DW), followed by parishin A (2.03 ± 0.05–4.86 ± 0.20 mg/g DW) and parishin E (2.16 ± 0.01–3.24 ± 0.03 mg/g DW). Most previous studies on the bioactive compounds of wild and outdoor field-grown GER have reported 4-HBA, gastrodin, and parishin derivatives, particularly parishin A, parishin B, and parishin C, as the major constituents [25,26]. However, in GER cultivated in Korea, *p*-HBME content was markedly higher under both cultivation methods. Yang et al. analyzed GER collected from Zhaotong county in Yunnan province, China, by HPLC and detected a prominent *p*-HBME peak, noting that its abundance varied depending on drying and heating conditions [41]. With respect to the pharmacological activity of *p*-HBME, only one study has demonstrated its potential to enhance learning and memory in mice in vivo [43], and more comprehensive investigations on its biological activities have not yet been conducted. Therefore, further studies employing GER samples rich in *p*-HBME, as well as purified *p*-HBME, are required to elucidate its potential pharmacological applications.

In mature GER, the total amount of the seven identified compounds was 12.15 ± 0.14 mg/g DW in IFC, whereas the three samples (OF-Y, OF-L, and OF-K) ranged from 13.21 ± 0.06 to 18.33 ± 0.05 mg/g DW. In contrast, the proportion of *p*-HBME within the total phenolic compounds was 40.73 ± 0.54% in IFC, whereas among the three samples (OF-Y, OF-L, and OF-K) it ranged from 35.28 ± 0.32% to 36.67 ± 0.13%, indicating a higher relative abundance of p-HBME in GER cultivated by the IFC method. This trend is likely attributable to the lower content of parishin derivatives in IFC-cultivated GER compared with OFC-cultivated GER. Further investigation is required to clarify why *p*-HBME levels in Korea-cultivated GER are higher than those reported for Chinese GER. In this study, the contents of gastrodin and 4-HBA, both used as quality control indicators for crude GER drugs, were lower than the values previously reported for both Chinese and Korean GER.

For example, Kim and Park [45] reported that the gastrodin content in GER cultivated in three regions of Korea ranged from 0.3 to 0.6 mg/g DW. Ha et al. reported that GER produced in the Muju region, a major production area for G. elata in Korea, contained 572.51 ± 14.68 μg/g DW of gastrodin and 4293.33 ± 117.23 μg/g DW of 4-HBA [46]. Kang et al. further reported that dried Korean GER contained 0.178% gastrodin and 0.376% 4-HBA on a dry-weight basis [47]. In GER produced in the Gansu region of China, gastrodin and 4-HBA contents were 0.119% and 0.129%, respectively, whereas GER produced in Tibet contained 0.177% gastrodin and 0.444% 4-HBA. Moreover, analysis of GER collected from eight regions in China showed that gastrodin levels ranged from 0.279 ± 0.040 to 0.485 ± 0.174%, 4-HBA ranged from 0.044 ± 0.016 to 0.097 ± 0.062%, and the sum of gastrodin and 4-HBA ranged from 0.359 to 0.541%. The Chinese Pharmacopoeia stipulates that the combined content of gastrodin and 4-HBA (gastrodigenin) must be ≥0.25% as a quality marker for GER, while the Korean Pharmacopoeia specifies a minimum of ≥0.20% for dried GER used as crude drug material. However, further investigation is needed to elucidate the changes in gastrodin and 4-HBA contents resulting from the hydrolysis of parishin derivatives and the possible formation of 4-HBA through demethylation of p-HBME during heat drying processes.

GER can be classified into mature and immature types based on growth stage, even when planted simultaneously and cultivated under identical conditions. As shown in Figure 2, mature GER is characterized by a large rhizome and the presence of developed flower buds, conferring high commercial value and making it the primary form used for medicinal and food purposes. In contrast, immature GER is smaller in size, lacks flower bud formation, and is mainly used as seed material for the following year’s cultivation. Although immature GER also has potential for medicinal and food applications, its chemical constituents have not yet been clearly elucidated. The phenolic compound profiles of mature and immature GER, according to growth stage, are presented in Table 4. Immature GER showed higher levels of p-HBME, parishin A, and parishin E than mature GER under both cultivation methods. Additionally, parishin B was abundant in OF-Y and OF-L, but was not detected in immature IFC or OF-K samples. The contents of parishin A and parishin E did not differ significantly between mature and immature GER produced by the IFC method. However, the p-HBME content was higher in immature GER (6.33 ± 0.09 mg/g DW) than in mature GER (4.95 ± 0.07 mg/g DW). These findings suggest that immature GER also has potential for medicinal or food applications.

#### 3.3.2. Effect of Harvest Time

The contents of gastrodin, its aglycone 4-HBA, and parishin derivatives vary not only with growth stage but also with harvest time, as these constituents change throughout the GER growth period [48,49]. In Korea, mature GER is generally cultivated for two years and harvested either in spring (April) or autumn (October–November), suggesting the possibility of seasonal variation in bioactive compound composition; however, relevant information remains limited. Morphological traits, yield per unit area, and the composition of GER constituents are also known to differ according to harvest time. The phenolic compound profiles according to harvest season are summarized in Table 5. Among the quantified compounds, 4-HBA and parishin C exhibited low contents and did not differ significantly between spring- and autumn-harvested GER. *p*-HBME content was slightly higher in the autumn-harvested GER than in spring-harvested GER. In contrast, the contents of parishin E, parishin B, and parishin A were significantly higher in the spring-harvested GER than those of autumn-harvested GER. A previous study on harvest time effects in GER grown in the same field reported that gastrodin content in autumn-harvested GER was approximately 1.6-fold higher than in spring-harvested GER. The same study also reported that vanillyl alcohol showed no significant seasonal difference [45]. Considering the life cycle of *G. elata*, growth begins around May after planting, when the rhizome establishes a symbiotic association with *Armillaria* sp., and continues until October, during which immature GER develops. Growth is inhibited during winter and resumes the following spring once temperatures return to the optimal range. This seasonal growth cycle is considered to influence the composition of bioactive constituents in GER.

The total content of the seven phenolic compounds was 20.67 ± 0.17 mg/g DW in spring-harvested GER, which was 1.72-fold higher than that of autumn-harvested GER (12.01 ± 0.19 mg/g DW). However, the proportion of *p*-HBME within the total phenolics was 23.14 ± 0.07% in spring-harvested GER and 48.91 ± 0.47% in autumn-harvested GER, indicating a 2.11-fold increase in autumn-harvested samples. Although *p*-HBME contains a methyl group attached to the primary alcohol of the 4-HBA backbone, its biological activities in vitro and in vivo remain largely unknown. These results may provide useful information for determining the optimal harvest time for GER.

### 3.4. Total Phenol and Total Flavonoid Contents

#### 3.4.1. Effect of Cultivation Method and Growth Stage

**Figure 5:**
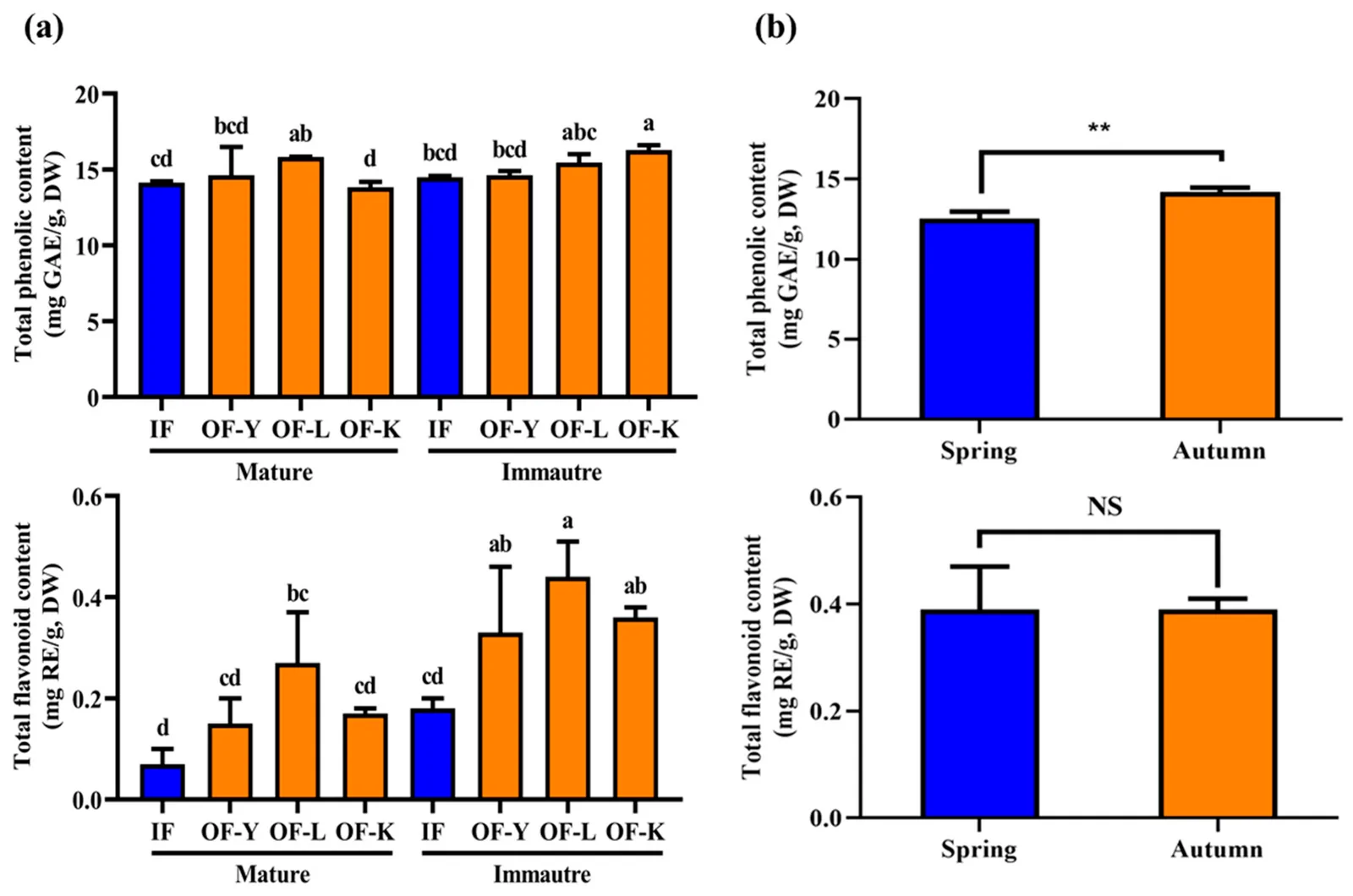
Comparison of total phenolic and flavonoid contents of *Gastrodia elata* rhizomes (GERs) according to cultivation method, growth stage, and harvest season. (**a**) Comparison of total phenolic content (TPC) and total flavonoid content (TFC) in mature and immature GER cultivated under indoor facility cultivation (IFC) and outdoor field cultivation (OFC). IF, indoor facility; OF-Y, L and K denote different outdoor farms. Data are presented as the mean ± SD (*n* = 3). Different lowercase letters (a–d) indicate significant differences according to Duncan’s multiple range test (*p* < 0.05). (**b**) Comparison of TPC and TFC between spring and autumn harvests. Data are presented as the mean ± SD (*n* = 3). Statistical significance was determined using Student’s *t*-test. (** *p* < 0.01; NS, not significant).

Plants or plant-based products contain various phytochemicals, including polyphenolics, carotenoids, alkaloids, saponins, and nitrogen- or sulfur-containing compounds, which exhibit diverse biological activities both in vitro and in vivo. As secondary metabolites of plants, phenolic acids and flavonoids, which belong to the polyphenol class, are well known for their antioxidant activity, and the compositions and contents of these phenolic compounds vary among plant species [50,51]. The results of comparisons of the TPC and TFC of GER produced by the IFC and OFC methods are presented in Figure 5a. The TPC of mature GER produced by the IFC method was 14.14 ± 0.07 mg GAE/g DW, whereas that of the three mature samples produced by the OFC method ranged from 13.82 ± 0.36 to 15.82 ± 0.02 mg GAE/g DW, showing no significant differences between cultivation methods.

In contrast, TFC was significantly higher in GER produced by the OFC method (0.15 ± 0.05–0.27 ± 0.06 mg RE/g DW) than in GER produced by the IFC method (0.07 ± 0.03 mg RE/g DW), and it was also higher in immature GER (0.18 ± 0.02–0.44 ± 0.07 mg RE/g DW) than in mature GER (0.07 ± 0.03–0.27 ± 0.06 mg RE/g DW). Moreover, the TFC contents of GER produced by OFC across the three cultivation sites showed significant variations between mature and immature samples. However, no significant differences were observed between mature and immature GER when compared solely by growth stage. Furthermore, the TFC of GER produced by the IFC method was lower than that of GER produced by the OFC method, with this tendency being more pronounced in immature GER. Lee et al. reported that steamed GER produced in Korea contained 9.07 ± 0.51 mg GAE/g of total phenols and 0.59 ± 0.09 mg RE/g of total flavonoids [52]. In comparison, the TPC observed in this study was higher, whereas the TFC was lower than previously reported values. These discrepancies are likely attributable to differences in the cultivation environments of the GER.

#### 3.4.2. Effect of Harvest Time

The effects of harvest time on TPC and TFC were investigated, and the results are presented in Figure 5b. TPC was significantly higher (*p* < 0.01, t-value; −5.624) in samples harvested in autumn (14.19 ± 0.44 mg GAE/g DW) compared with spring-harvested GER (12.53 ± 0.44 mg GAE/g DW). In contrast, TFC showed little variation between the two harvest times, with no significant differences observed.

These results are likely attributable to the fact that most phenolics in GER consist of benzene-based compounds substituted with hydroxyl groups, whereas the content of flavonoids possessing phenylpropanoid skeletons is relatively low. GER is particularly rich in hydroxyl-substituted phenolic compounds. Of the approximately 630 constituents identified in GER to date, 227 are classified as aromatic compounds, including 45 monobenzyl compounds, 28 parishin derivatives, and 19 aromatic substituted glycosides [12]. These aromatic constituents are expected to contribute substantially to the overall antioxidant activities in GER.

### 3.5. Antioxidant Activity

#### 3.5.1. Effect of Cultivation Method and Growth Stage

**Figure 6:**
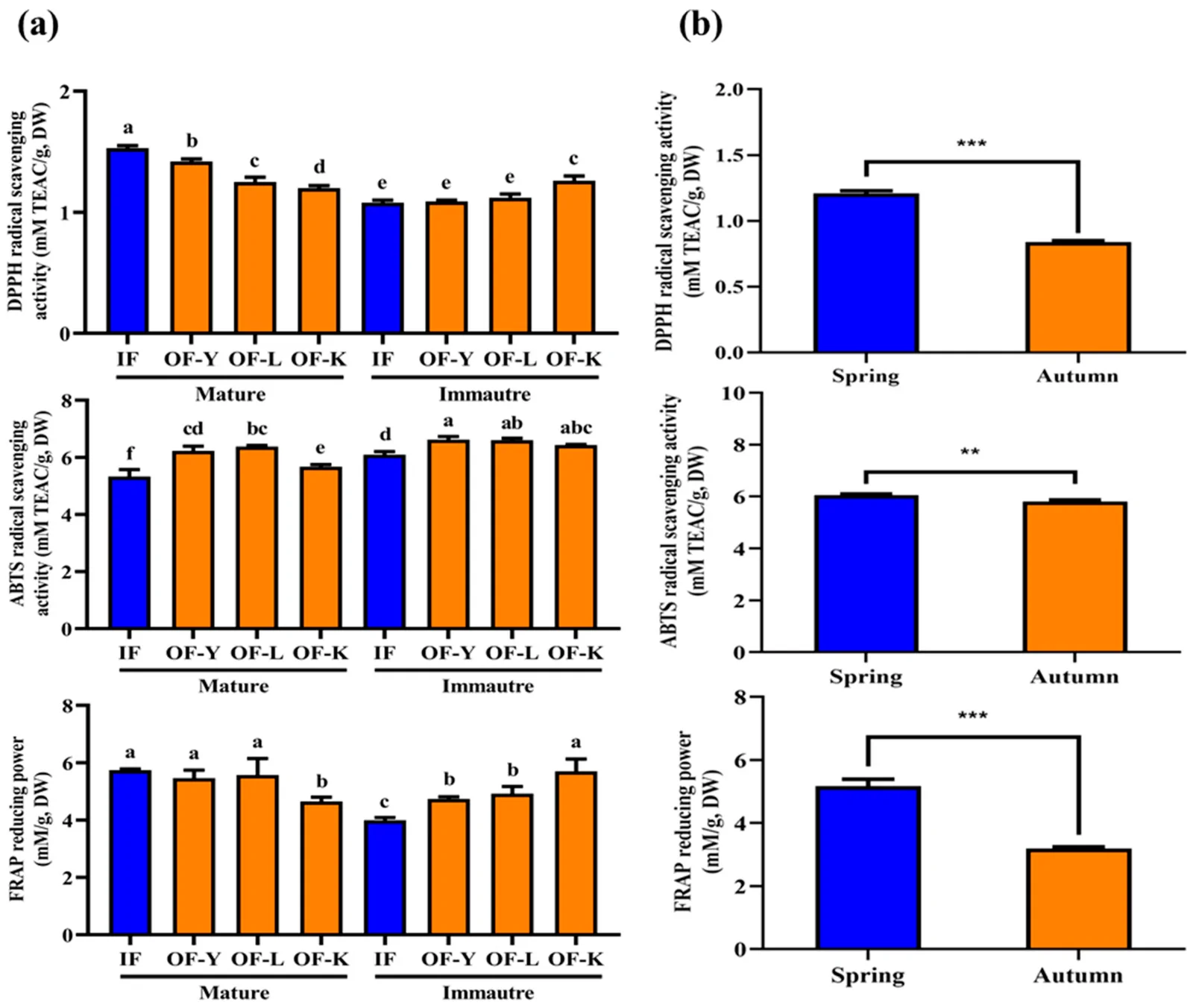
Comparison of antioxidant activities of *Gastrodia elata* rhizomes (GERs) according to cultivation method, growth stage, and harvest time. (**a**) Comparison of antioxidant activities in mature and immature GER produced by indoor facility cultivation (IFC) and outdoor field cultivation (OFC). IF, indoor facility; OF, outdoor field; Y, L, and K denote different farms. Values are expressed as mg/g DW and presented as mean ± SD (*n* = 3). Different superscript letters (a–f) indicate significant differences according to Duncan’s multiple range test (*p* < 0.05). (**b**) Comparison of antioxidant activities of GER at different harvest times. Values are presented as mean ± SD (*n* = 3). (** *p* < 0.01, *** *p* < 0.001).

Plant extracts or plant-derived products exhibit antioxidant activity through various chemical reaction mechanisms. Therefore, a single assay method is insufficient to fully characterize the antioxidant properties of target materials [53]. Consequently, it is recommended to employ at least two complementary assay methods to investigate their antioxidant properties. Various analytical methods have been applied depending on the nature of the bioactive compounds present in the samples. Among various methods, DPPH and ABTS radical scavenging activities and FRAP reducing power assay are widely used to evaluate the antioxidant capacities of natural products and foods [34]. GER contains phenolic compounds with diverse chemical structures, and these constituents play an important role in its antioxidant activity [54]. The results obtained from three different methods to compare the antioxidant activities of mature and immature GER cultivated using two systems (IFC and OFC) are presented in Figure 6a. The DPPH radical scavenging activity of mature GER produced by the IFC method (1.53 ± 0.02 mM TEAC/g DW) was significantly higher (*p* < 0.05) than those of the three mature samples produced by the OFC method (1.10 ± 0.02–1.42 ± 0.02 mM TEAC/g DW). The three OFC-derived mature samples also exhibited significant differences in DPPH activity among farm locations. These variations are likely attributed to differences in fertilization and irrigation practices. In immature GER, the DPPH radical scavenging activities of the IFC samples and two OFC samples (OFY and OF-L), excluding OF-K, did not differ significantly. When compared by growth stage, DPPH activity was significantly higher (*p* < 0.05) in mature GER (1.20 ± 0.02–1.53 mM TEAC/g DW) than in immature GER (1.09 ± 0.01–1.26 ± 0.04 mM TEAC/g DW) across both cultivation methods.

When comparing ABTS activity according to cultivation method and growth stage, GER produced by the OFC method showed higher activity (6.09 ± 0.11–6.62 ± 0.11 mM TEAC/g DW) than that of the IFC method (5.33 ± 0.24–6.38 ± 0.04 mM TEAC/g DW). ABTS activity was also significantly higher (*p* < 0.05) in immature GER than in mature GER, showing a consistent pattern across cultivation methods. In contrast, FRAP activity was generally higher in mature GER than in immature GER.

GER is known to contain substantial amounts of parishin derivatives and their hydrolysis products, gastrodin and 4-HBA, which play crucial roles in its pharmacological activities. Although 4-HBA, a major active compound of GER, has been reported to reduce oxidative stress and exhibit strong antioxidant activity in vivo [15], p-HBME has also been identified as a bioactive phenolic constituent with pharmacological potential, including neuroprotective effects [52]. To date, approximately 630 chemical constituents have been identified in raw GER and its processed products, about 70% of which are phenolic compounds. Many of these phenolic constituents exhibit antioxidant properties [12]. Accordingly, the antioxidant activity of GER is believed to be associated with the presence of parishin derivatives and their metabolites.

#### 3.5.2. Effect of Harvest Time

In Korea, GER is cultivated for two years per cycle and harvested in spring (April) or autumn (October) as needed. To investigate the effect of harvest time on GER’s antioxidant activity, the results obtained from three assay methods are shown in Figure 6b. DPPH and FRAP activities were significantly higher (*p* < 0.001) in samples harvested in spring than in those harvested in autumn. ABTS activity was also significantly higher in spring-harvested samples (*p* < 0.01). As shown in Table 5, the total amount of the seven phenolic compounds was 12.01 ± 0.19 mg/g DW in GER harvested in autumn, whereas it was 20.67 ± 0.17 mg/g DW in GER harvested in spring, representing a 1.72-fold increase. This difference in antioxidant activity is likely attributed to variations in phenolic compound content. These results suggest that GER can serve as an effective antioxidant source. In addition to phenolic compounds, GER is known to contain other antioxidant constituents, including polysaccharides and ergothioneine, both of which have also been reported to exert antioxidant activity. Therefore, further studies are required to clarify the individual contributions of these constituents to the overall antioxidant activity of GER and to examine potential synergistic interactions among them.

#### 3.5.3. Correlation Between Antioxidant Activities and Bioactive Compounds

**Figure 7:**
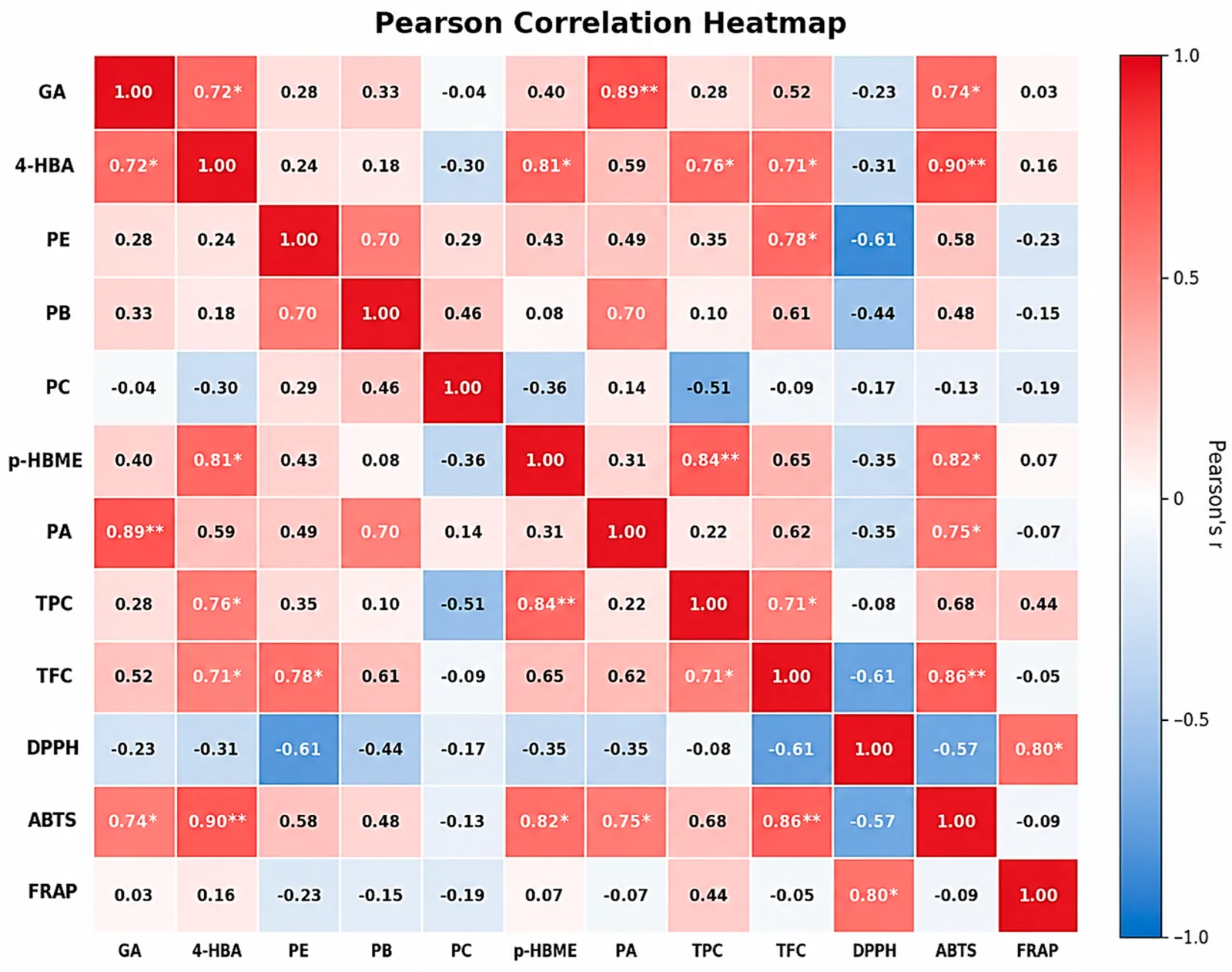
Pearson correlation heatmap showing the relationships among individual phenolic compounds, total phenolic content (TPC), total flavonoid content (TFC), and antioxidant activities (DPPH, ABTS, and FRAP) of GER samples. Values in each cell represent Pearson’s correlation coefficients (r). * *p* < 0.05, ** *p* < 0.01.

**Figure 8:**
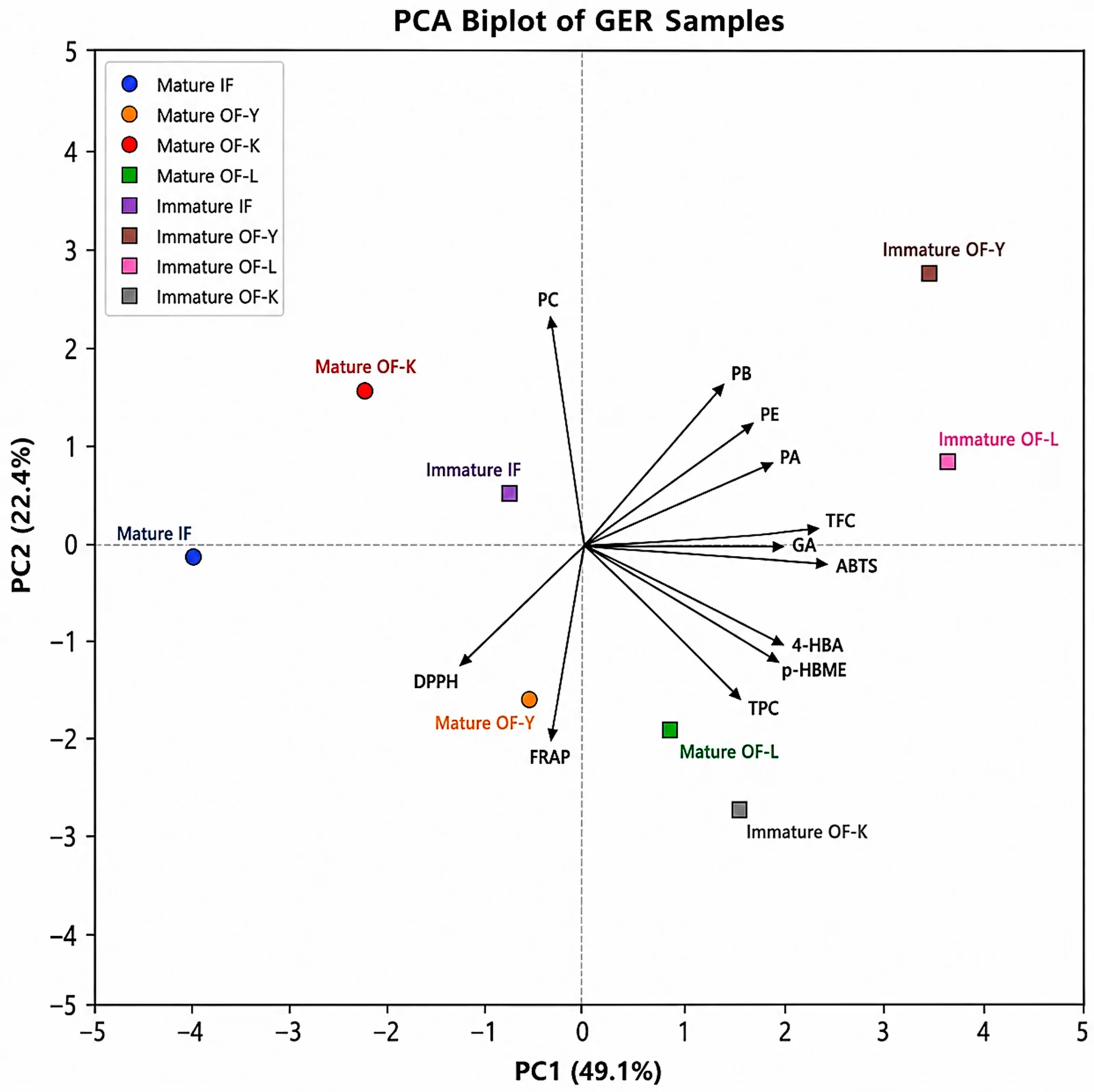
Principal component analysis (PCA) biplot of GER samples based on individual phenolic compounds, total phenolic content (TPC), total flavonoid content (TFC), and antioxidant activities. PC1 and PC2 explained 49.1% and 22.4% of the total variance, respectively, with a cumulative explained variance of 71.5%.

Pearson’s correlation analysis was performed to evaluate the relationships among individual phenolic compounds, total phenolic content (TPC), total flavonoid content (TFC), and three antioxidant activities [Figure 7]. ABTS radical scavenging activity showed significant positive correlations with gastrodin (r = 0.74, *p* < 0.05), 4-HBA (r = 0.90, *p* < 0.01), p-HBME (r = 0.82, *p* < 0.05), PA (r = 0.75, *p* < 0.05), and TFC (r = 0.86, *p* < 0.01). In contrast, no significant correlations were observed between individual phenolic compounds and DPPH or FRAP activities. Among the antioxidant assays, DPPH and FRAP showed a significant positive correlation (r = 0.80, *p* < 0.05). These results indicate that the relationships between phenolic composition and antioxidant activity may vary depending on the antioxidant assay, with ABTS activity being more closely associated with variations in several phenolic constituents. PCA was further performed to characterize the overall variation in phenolic composition and antioxidant properties among GER samples [Figure 8]. PC1 and PC2 accounted for 49.1% and 22.4% of the total variance, respectively, explaining 71.5% of the cumulative variance. The samples showed distinct distributions according to growth stage and cultivation conditions. In particular, Immature OF-Y and OF-L were positioned mainly on the positive side of PC1 and showed similar directional associations with PE, PB, PA, TFC, and ABTS. In contrast, Mature IF was clearly separated from the other samples along the negative direction of PC1, while Mature OF-Y and Immature OF-K were differentiated mainly along PC2. These results indicate that differences in phenolic composition and antioxidant properties associated with growth stage and cultivation conditions contributed to the discrimination among GER samples.

## 4. Discussion

GER has long been used as a traditional herbal medicine and health food ingredient in East Asia. Among the bioactive constituents of GER, phenolic compounds have attracted the greatest attention, and their contents and compositions are known to be affected by cultivation practices and post-harvest processing. This study compared the profiles of major phenolic compounds and antioxidant activities of GER according to cultivation methods (IFC and OFC), growth stages (mature and immature), and harvest seasons (spring and autumn). Quantitative analysis of seven phenolic compounds revealed that p-HBME, parishin A, parishin B, and parishin E were consistently present as major constituents regardless of cultivation method. Notably, p-HBME, previously reported only as a trace component in GER, was characteristically abundant in Korean GER analyzed in this study. In contrast, gastrodin and 4-HBA, both widely used as quality control markers, were detected at relatively low levels. Gastrodin and 4-HBA have similarly been highlighted as key quality control markers in a recent co-extraction study on GER [55], whereas the characteristic abundance of p-HBME observed in this study may warrant its consideration as an additional candidate marker for Korean-grown GER. Comparison of IFC and OFC samples suggested that IFC-produced GER contained lower amounts of parishin A, parishin B, and 4-HBA than OFC-produced GER. Although the total content of the seven quantified compounds was lower in IFC-produced GER, the proportion of p-HBME was relatively higher than that of OFC-produced GER, which is likely attributable to the relatively low levels of parishin derivatives in IFC-produced GER. Furthermore, immature GER contained higher TPC than mature GER, and GER harvested in spring exhibited higher total phenolic levels than those harvested in autumn. Although mature-type GER with developed flower buds is traditionally regarded as high-quality, immature GER also demonstrated considerable potential for commercial utilization as a medicinal herb or food material. As indicators of antioxidant capacity, both DPPH radical scavenging activity and FRAP reducing power were higher in IFC-produced GER than in OFC-produced GER and tended to be higher in mature GER than in immature GER. With respect to harvest season, all antioxidant activity indicators were higher in GER harvested in spring than in autumn.

GER has long been used as a medicinal herb and is also employed as a raw material in general foods and health functional foods. Therefore, the findings of this study provide foundational information for optimizing cultivation methods and improving the quality control and utilization of GER produced in Korea. However, because IFC and OFC samples were obtained from different cultivation regions, the observed differences may have been influenced not only by cultivation method but also by regional environmental factors, including soil characteristics and microclimate. This should be considered a limitation of the present study. Future studies should directly compare IFC and OFC at the same cultivation site to minimize the influence of regional environmental variation, thereby enabling a clearer distinction between the effects of cultivation method and those of regional environmental variation. In addition, studies involving a broader range of cultivation environments are needed to further investigate the relationships between cultivation conditions, symbiotic microorganisms, and phytochemical characteristics.

## 5. Conclusions

This study demonstrated that cultivation method, growth stage, and harvest season were associated with variations in the phenolic composition and antioxidant properties of *Gastrodia elata* rhizome (GER). Notably, Korean-grown GER was characterized by a high abundance of *p*-hydroxybenzyl methyl ether (*p*-HBME), suggesting its potential as a characteristic phytochemical component that warrants further investigation. Spring-harvested GER showed higher levels of several individual phenolic compounds and generally stronger antioxidant activities than autumn-harvested GER, whereas TPC was higher in the autumn-harvested samples. However, the seasonal comparison should be interpreted cautiously because the analytical replicates were derived from the same biological sample. Immature rhizomes also exhibited substantial phenolic contents and antioxidant activities, suggesting their potential for further investigation as medicinal and functional food resources; however, as immature samples were available only from the spring 2025 harvest, the growth-stage comparison and the harvest-season comparison were based on different sample sets and are therefore not fully parallel. Because the IFC and OFC samples were obtained from different cultivation regions, the observed differences cannot be attributed solely to cultivation method and may also reflect regional environmental factors, including soil characteristics and microclimate. These findings provide a basis for further investigation of cultivation and harvest conditions associated with the phytochemical characteristics and antioxidant properties of Korean GER. Future studies using independent biological replicates and directly comparing IFC and OFC under the same regional conditions are needed to distinguish cultivation effects from environmental variation and to clarify the relationships among cultivation conditions, symbiotic microorganisms, and phytochemical characteristics.

## Figures and Tables

**Table 1:** List of GER plants used in this study.

Sample No.	Cultivation Method	Harvest Year	Harvest Season	Growth Stage	Cultivation Region
1	Indoor facility	2024	Spring	Mature	Jinan ^1^
2	Indoor facility	2024	Autumn	Mature	Jinan
3	Indoor facility	2025	Spring	Mature	Jinan
4	Outdoor field-Y ^3^	2025	Spring	Mature	Muju ^2^
5	Outdoor field-L ^3^	2025	Spring	Mature	Muju
6	Outdoor field-K ^3^	2025	Spring	Mature	Muju
7	Indoor facility	2025	Spring	Immature	Jinan
8	Outdoor field-Y	2025	Spring	Immature	Muju
9	Outdoor field-L	2025	Spring	Immature	Muju
10	Outdoor field-K	2025	Spring	Immature	Muju

^1^ Medicinal Resource Research Institute under Jeonbuk Agricultural Research and Extension Services, Jinan-eup (Jinan-gun, Jeonbuk, Republic of Korea). ^2^ Ansung-myeon (Muju-gun, Jeonbuk, Republic of Korea). ^3^ Outdoor field-Y, -L and -K are cultivated at three different farms in same region.

**Table 2:** Calibration curve, coefficient of determination and linear ranges of seven phenolic compounds.

Compound	UVmax (nm)	Calibration Curve	R^2^	Linear Range (µg/mL)
4-HBA	220.6, 272.5	y = 31002x + 103784	0.9996	0.9766–500
Gastrodin	218.5, 268.9	y = 25090x + 31187	0.9999	3.906–500
Parishin E	220.6, 268.9	y = 6272.5x − 98977	0.9999	3.906–500
Parishin B	220.6, 268.9	y = 12736x − 114426	0.9997	5.8594–750
Parishin C	220.6, 268.9	y = 13388x − 149839	0.9996	6.8359–875
p-HBME	223.0, 272.5	y = 29633x + 68891	0.9999	0.9766–250
Parishin A	218.3, 268.9	y = 15885x − 170247	0.9996	6.25–800

4-HBA, 4-hydroxybenzyl alcohol; PE, parishin E; PB, parishin B; PC, parishin C; p-HBME, p-hydroxybenzyl methyl ether; PA, parishin A.

**Table 3:** Chemical shifts in compound 1 and 2.

Position ^1^	1 (δ, ppm)		2 (δ, ppm)		
	^13^C-NMR	^1^H-NMR	^13^C-NMR	^1^H-NMR	DEPT
1	133.4		129.08		C
2	129.9	7.17 (d, 2H, *J* = 8.4)	130.79	7.12 (d, 2H, *J* = 9.0)	CH
3	116.1	6.75 (d, 2H, *J* = 8.4)	116.24	6.73 (d, 2H, *J* = 8.4)	CH
4	158.0		158.33		C
5	116.1	6.75 (d, 2H, *J* = 8.4)	116.24	6.73 (d, 2H, *J* = 8.4)	CH
6	129.9	7.17 (d, 2H, *J* = 8.4)	130.79	7.12 (d, 2H, *J* = 9.0)	CH
7	65.1	4.48 (s, 2H)	75.53	4.30 (s, 2H)	CH_2_
8			57.79	3.29 (s, 3H)	CH_3_

^1^ The atom numbering corresponding to the positions is shown in Figure 4.

**Table 4:** Content of phenolic compounds in mature and immature GER produced by IFC and OFC methods.

Sample	GA	4-HBA	PE	PB	PC	*p*-HBME	PA	Total (mg g^−1^) ^1^	*p*-HBME/ Total (%)
Mature									
IF	0.19 ± 0.01 ^f^	0.85 ± 0.01 ^f^	3.44 ± 0.05 ^d^	1.12 ± 0.03 ^d^	0.35 ± 0.01 ^c^	4.95 ± 0.07 ^f^	1.25 ± 0.01 ^h^	12.15 ± 0.14	40.73 ± 0.54
OF-Y	1.37 ± 0.02 ^a^	1.76 ± 0.00 ^b^	2.16 ± 0.01 ^g^	ND	ND	5.88 ± 0.02 ^e^	4.86 ± 0.20 ^c^	16.02 ± 0.19	36.67 ± 0.13
OF-L	0.53 ± 0.01 ^d^	1.77 ± 0.02 ^b^	3.24 ± 0.03 ^e^	2.91 ± 0.14 ^b^	ND	6.65 ± 0.09 ^b^	3.24 ± 0.03 ^d^	18.33 ± 0.05	36.27 ± 0.48
OF-K	0.39 ± 0.10 ^e^	1.22 ± 0.01 ^e^	2.74 ± 0.19 ^f^	1.75 ± 0.05 ^c^	0.41 ± 0.00 ^b^	4.66 ± 0.04 ^g^	2.03 ± 0.05 ^f^	13.21 ± 0.06	35.28 ± 0.32
Immature									
IF	0.46 ± 0.02 ^de^	1.34 ± 0.04 ^d^	3.78 ± 0.07 ^c^	ND	ND	6.33 ± 0.09 ^c^	1.81 ± 0.04 ^g^	13.72 ± 0.26	46.16 ± 0.68
OF-Y	1.12 ± 0.05 ^b^	1.68 ± 0.02 ^c^	5.42 ± 0.03 ^a^	5.08 ± 0.06 ^a^	0.83 ± 0.03 ^a^	6.34 ± 0.07 ^c^	5.69 ± 0.18 ^b^	26.16 ± 0.18	24.22 ± 0.29
OF-L	1.16 ± 0.03 ^b^	1.66 ± 0.01 ^c^	5.52 ± 0.05 ^a^	5.13 ± 0.09 ^a^	ND	6.14 ± 0.02 ^d^	6.13 ± 0.06 ^a^	25.74 ± 0.16	23.87 ± 0.09
OF-K	0.77 ± 0.02 ^c^	1.89 ± 0.01 ^a^	4.27 ± 0.04 ^b^	ND	ND	6.96 ± 0.03 ^a^	2.23 ± 0.01 ^e^	16.11 ± 0.09	43.17 ± 0.20

All samples were harvested in the spring of 2025. IF, indoor facility; OF, outdoor field. OF-Y, L and K, outdoor field cultivation at three different farms in the same region. GA, gastrodin; 4-HBA, 4-hydroxybenzyl alcohol (gastrodigenin); P, parishin; p-HBME, p-hydroxybenzyl methyl ether. All values (mg/g DW) are mean ± SD of three extraction replicates (*n* = 3) obtained from a composite sample pooled from three individual rhizomes per condition. ^1^ Sum of seven phenolic compounds as a dry weight base in Table 4. ND, not detected. Different superscript letters (a–h) in the same column indicate significant differences according to Duncan’s multiple range test (*p* < 0.05; a > d).

**Table 5:** Impact of harvest time on phenolic compounds of the mature GER produced by IFC.

Harvest Time	GA	4-HBA	PE	PB	PC	*p*-HBME	PA	Total (mg g^−1^)	*p*-HBME/ Total (%)
Spring	0.59 ± 0.01	1.36 ± 0.01	3.50 ± 0.05	3.32 ± 0.08	0.58 ± 0.01	4.78 ± 0.01	6.54 ± 0.04	20.67 ± 0.17	23.14 ± 0.07
Autumn	ND	1.51 ± 0.08	1.81 ± 0.03	0.95 ± 0.02	0.34 ± 0.01	5.88 ± 0.05	1.54 ± 0.03	12.01 ± 0.19	48.91 ± 0.47
*t*-Value	144.52 ***	−3.37 *	53.29 ***	52.56 ***	22.22 ***	−37.03 ***	175.28 ***		

The GER samples were cultivated in the same indoor facility and harvested in the spring (April) and autumn (October) of 2024 after 2 years of cultivation. The sum of seven phenolic compounds as a dry weight base by HPLC. ND, not detected. GA, gastrodin; 4-HBA, 4-hydroxybenzyl alcohol; P, parishin; p-HBME, p-hydroxybenzyl methyl ether. All values (mg/g DW) are presented as the mean ± SD of four extraction replicates (*n* = 4) obtained from a composite sample pooled from four individual rhizomes per harvest season. (*** *p* < 0.001, * *p* < 0.05).

## Data Availability

The original contributions presented in this study are included in the article. Further inquiries can be directed to the corresponding author.

## Abbreviations

The following abbreviations are used in this manuscript:

GER	*Gastrodia elata* rhizome
OFC	Outdoor field cultivation
IFC	Indoor facility cultivation
DW	Dry weight basis
HPLC	High performance liquid chromatography
UV-Vis	Ultraviolet-visible spectroscopy
GC-MS	Gas chromatography-mass spectrometry
GA	Gastrodin
4-HBA	4-hydroxybenzyl alcohol
*p*-HBME	*p*-hydroxybenzyl methyl ether
PE	Parishin E
PB	Parishin B
PC	Parishin C
PA	Parishin A
UPLC-QTOF-MS	Ultra-performance liquid chromatography-quadrupole time-of-flight mass spectrometry
NMR	Nuclear magnetic resonance
SCC	Silica gel column chromatography
TPC	Total phenol content
TFC	Total flavonoid content
GAE	Gallic acid equivalent
RE	Rutin equivalent
DPPH	2,2-diphenyl-1-picrylhydrazyl
ABTS	2,2′-azino-bis(3-ethylbenzothiazoline-6-sulfonic acid)
FRAP	Ferric reducing antioxidant power
TPTZ	2,4,6-tripyridyl-*s*-triazine
Trolox	6-hydroxy-2,5,7,8-tetramethylchroman-2-carboxylic acid
TEAC	Trolox equivalent antioxidant capacity
PCA	Principal component analysis
